# Supplementary figures and images for: Accelerated straw decomposition and mitigated methane emissions via autumn puddling incorporation enhances soil health and yield stability in cold-region rice systems of China
Source: PeerJ. 2025 Oct 27;13:e20264. doi: 10.7717/peerj.20264 (PMC12574589; doi:10.7717/peerj.20264)

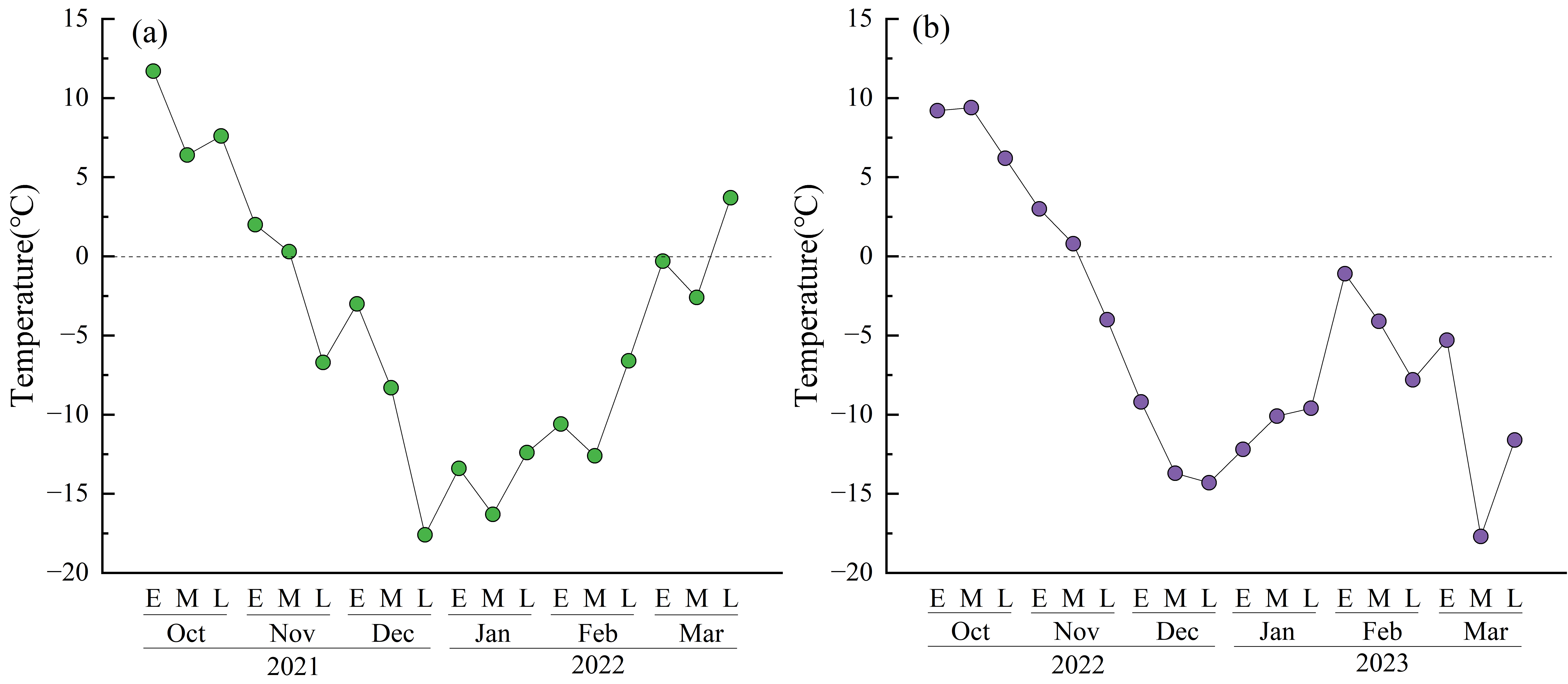

Supplement: Supplemental Information 2 — (E) Early part of the month. (M) Mid part of the month. (L) Late part of the month. [file peerj-13-20264-s002.png]
